# A Multidimensional Disease Weighted Index (DWI) for Risk Assessment in COPD: A Proposed Conceptual Framework and Pre-Specified Validation Roadmap

**DOI:** 10.3390/jcm15218335

**Published:** 2026-10-26

**Authors:** Alberto Ricci, Daniel Piamonti, Bruno Sposato, Alessandra Pagliuca, Raffaele Sglavo, Pier-Valerio Mari, Michela D’Ascanio

**Affiliations:** 1 AOU Sant’Andrea Hospital, 00189 Rome, Italy; alberto.ricci@uniroma1.it (A.R.); daniel.piamonti@uniroma1.it (D.P.); a.pagliuca90@gmail.com (A.P.); raffaele.sglavo@gmail.com (R.S.); dascaniomichela87@gmail.com (M.D.); 2 Clinical and Molecular Medicine Department, Sapienza University of Rome, 00185 Rome, Italy; 3 Pneumology Department, Local Health Unit ‘Toscana Sud-Est’, ‘Misericordia’ Hospital, 58100 Grosseto, Italy; bruno.sposato@uslsudest.toscana.it; 4 Internal Medicine Unit, San Carlo di Nancy Hospital, GVM Care & Research, 00165 Rome, Italy

**Keywords:** COPD, chronic obstructive pulmonary disease, risk stratification, multidimensional assessment, treatable traits, biomarkers, NT-proBNP, Disease Weighted Index, comorbidities, prognosis

## Abstract

**Background/Objectives**: Chronic obstructive pulmonary disease (COPD) is a heterogeneous condition whose prognosis is shaped by exacerbations and by cardiovascular, inflammatory and metabolic comorbidity, which staging based on forced expiratory volume in one second (FEV_1_) captures poorly. In this concept paper, we propose a conceptual multidimensional prognostic index, the Disease Weighted Index (DWI), together with a pre-specified programme for its derivation and validation; the work reports no model development or validation. **Methods**: Ten routinely available parameters were grouped into four domains: Pulmonary–Symptomatic (FEV_1_, COPD Assessment Test [CAT] score, exacerbation history), Cardio–Systemic (N-terminal pro-B-type natriuretic peptide [NT-proBNP], C-reactive protein, New York Heart Association [NYHA] class), Metabolic–Nutritional (glycated haemoglobin [HbA1c], body mass index) and Demographic–Exposure (age, smoking history). Each parameter was scored on a three-level ordinal scale using guideline-based cut-offs and assigned a provisional, expert-informed weight through an explicit ordinal procedure in which the authors allocated it to a tier by judgement, guided by the adjusted effect estimates reported in the literature; these weights are specifications to be re-estimated, not established prognostic contributions. The weighted sum was normalised to a 0–100 scale. No patient-level data were used. **Framework**: NT-proBNP received the highest provisional weight (0.16). Four provisional risk categories (low, moderate, high and very high) were defined and linked to an expert opinion-based follow-up flowchart intended for prospective testing only. The pre-specified prediction target is a 12-month composite of severe exacerbation, all-cause hospitalisation or all-cause death. Rules for scoring when parameters are unavailable, worked examples, a conceptual comparison with the BODE, ADO, DOSE, CODEX and COTE indices, and a three-phase derivation and validation roadmap aligned with the TRIPOD statement are provided. **Conclusions**: The DWI is a pre-validation, testable framework that integrates the pulmonary and systemic dimensions of COPD. It is a research protocol for an index, not a validated clinical tool: its predictive performance is unproven, and it should not guide individual patient management until derivation and validation, including comparison with the unweighted sum of its items and with established indices, have been completed.

## 1. Introduction

Chronic Obstructive Pulmonary Disease (COPD) is a major cause of morbidity and mortality, frequently complicated by comorbidities—particularly cardiovascular disease (CVD)—that substantially influence prognosis [1,2,3,4,5]. Although genetic susceptibility, smoking, occupational exposures, and air pollution contribute to COPD development and progression, outcomes are increasingly shaped by exacerbations, hospitalisations, and systemic involvement [3,6]. Current stratification systems such as GOLD primarily rely on FEV_1_ and symptom burden [3]; however, these measures alone often fail to capture COPD heterogeneity and the extra-pulmonary factors that drive progression and adverse outcomes.

Exacerbations are pivotal events in the course of COPD, with implications for cardiopulmonary risk and for the choice of treatment [4,7], and their prevention is a central aim of maintenance inhaled therapy, including single-inhaler triple therapy, whose real-world effects on health status have been described [8]. Importantly, COPD is strongly associated with CVD (heart failure, ischemic heart disease, myocardial infarction, stroke, arrhythmias), due to shared risk factors and systemic inflammation [5,9,10,11]. CVD markedly worsens prognosis, with substantially higher cardiovascular mortality compared with matched individuals without COPD [12]. Exacerbations further amplify short-term cardiovascular risk and mortality [13,14,15,16]. Despite this interplay, cardiopulmonary risk is often under-recognised, and no widely adopted framework exists to stratify integrated cardiopulmonary risk in COPD.

Beyond heart–lung interactions, metabolic comorbidity—particularly diabetes—may further worsen COPD outcomes, yet remains less explored [17,18]. Diabetes is a leading cause of death [19] and shares pathogenic pathways with COPD and CVD, including chronic systemic inflammation and oxidative stress [20,21,22]. Diabetes is associated with a higher incidence of COPD [23] and is a frequent comorbidity in patients hospitalised for COPD, in whom its prognostic impact is not uniform [24], suggesting a potential interplay between COPD, CVD and metabolic dysfunction.

Several multidimensional indices have been developed to overcome the limitations of FEV_1_-based staging. The BODE index combines body mass index, airflow obstruction, dyspnoea and exercise capacity and predicts all-cause mortality [25]; the ADO index (age, dyspnoea, obstruction) has been related to the medical burden of COPD [26] and simplifies prognostic estimation for use in primary care [27]; the DOSE index (dyspnoea, obstruction, smoking, exacerbations) was designed to summarise overall disease severity and healthcare utilisation [28]; the CODEX index adds a weighted comorbidity term for patients discharged after an exacerbation [29]; and the COTE index quantifies the mortality contribution of twelve individual comorbidities [30]. Each of these instruments was derived and validated in patient cohorts, which is their decisive advantage over the framework proposed here. None of them, however, incorporates circulating biomarkers of cardiac strain, systemic inflammation or metabolic dysregulation, and none was constructed to be recomputed at every outpatient visit from data already present in the routine clinical record. Biomarkers such as NT-proBNP and C-reactive protein (CRP) may add predictive information by capturing subclinical cardiac strain and systemic low-grade inflammation, respectively. Accordingly, this study aimed to design a conceptual Disease Weighted Index (DWI) framework. By integrating traditional clinical status (BMI, NYHA class) and symptoms (CAT) with objective metabolic and inflammatory biomarkers (HbA1c, CRP, NT-proBNP), this proposed model is intended to be tested as a more comprehensive risk stratification than current survival indices; whether it achieves this, and whether it can inform clinical decisions, are questions for future derivation, validation and interventional studies. The present work is explicitly pre-empirical: it specifies the architecture of the index, the procedure used to derive its weights, the scoring rules, and the programme required to derive, calibrate and validate it in independent cohorts. No patient-level data were used at any stage.

## 2. Materials and Methods

The DWI was based on the selection of clinically meaningful parameters reflecting the multidimensional nature of COPD. Variables were chosen according to their documented association with exacerbations, hospitalisation, and mortality, as well as their availability in routine clinical practice. To ensure conceptual coherence, parameters were grouped into four predefined domains capturing Pulmonary–Symptomatic (FEV_1_ % predicted, exacerbations in the previous 12 months, CAT score), Cardio–Systemic (NT-proBNP, CRP, NYHA functional class), Metabolic–Nutritional (HbA1c, BMI) and Demographic–Exposure (age, smoking history) dimensions of the disease. All elements described in Section 2 and Section 3 were specified a priori and none were estimated from patient data; Section 3.5 distinguishes the elements fixed in the present work from those that the derivation and validation studies will establish empirically.

For each parameter, a brief evidence-based rationale is provided below, summarising its relevance in COPD and its proposed contribution to risk stratification.

### 2.1. FEV_1_

Forced expiratory volume in one second (FEV_1_) was included in the risk chart as the traditional marker of airflow limitation and structural pulmonary impairment in COPD. FEV_1_ has historically represented the cornerstone for disease classification and severity staging and remains a relevant indicator of long-term prognosis, as shown by its association with mortality [25] and by the natural history of FEV_1_ decline in smokers [31].

However, substantial evidence indicates that FEV_1_ alone provides an incomplete representation of disease burden and has limited ability to predict individual clinical trajectories, especially exacerbation risk [32] and survival [33]. For this reason, FEV_1_ was included in the present model as a core but non-exclusive prognostic component, intended to capture the static structural dimension of COPD rather than its dynamic or systemic manifestations.

In the DWI, FEV_1_ was assigned a provisional weight of 0.12 within the Pulmonary–Symptomatic domain. Its categories were aligned with the GOLD spirometric grades (≥ 80%, 50–79% and < 50% of predicted); FEV_1_ is intended to be measured after bronchodilator administration and expressed as percent predicted; the reference equations to be used, and the source and rationale of these thresholds, are detailed in Section 2.13.

### 2.2. Exacerbations

Exacerbations were included in the risk chart as one of the main clinical indicators of disease instability and progression. Among all variables evaluated, exacerbations represent one of the most potent predictors of COPD outcome, including recurrent and more frequent episodes of exacerbation, hospital admissions, and mortality [3]. Each exacerbation induces a transient worsening of airway and systemic inflammation, contributing to an accelerated decline in lung function and increasing the risk of cardiovascular disease. Furthermore, patients with recurrent exacerbations (usually defined as the “frequent exacerbator phenotype”) exhibit a characteristic inflammatory profile, characterised by higher airway inflammatory markers, such as sputum IL-6; moreover, recovery of lung function after an exacerbation is often slow or incomplete [34]. In the ECLIPSE cohort of 2138 patients, a history of exacerbations was the single best predictor of future exacerbations across all GOLD stages, consistent with a frequent-exacerbator phenotype that is independent of spirometric severity [35]. These findings justify the inclusion of exacerbations within the Pulmonary–Symptomatic domain, as they capture both the static component of obstruction (via FEV_1_) and the dynamic component of the disease.

A provisional weight of 0.08, corresponding to its tier in Table 1, was assigned to this parameter within the DWI. Exacerbation history is a strong but not exclusive predictor, since exacerbation is a consequence of multiple interacting mechanisms, attributable to airflow limitation, systemic inflammation, and comorbidities.

### 2.3. COPD Assessment Test (CAT)

The COPD Assessment Test (CAT) was included in the risk chart as a standardised patient-reported outcome measure reflecting symptom burden and health-related quality of life in COPD. The CAT score captures multiple dimensions of daily disease impact, including dyspnoea, cough, sputum production, chest tightness, activity limitation, sleep quality, and energy levels. Its development, measurement properties and minimal clinically important difference have been established [36,37,38].

Several studies have demonstrated that CAT provides prognostic information beyond FEV_1_, particularly in identifying patients with significant symptom burden despite moderate airflow limitation. CAT has also been shown to correlate with exacerbation frequency and to predict future exacerbations, making it a valuable marker of the symptomatic and clinical instability component of COPD [39,40]. Moreover, CAT is responsive to clinical changes and therapeutic interventions, supporting its role as a dynamic indicator of disease impact.

Within the present multidimensional framework, CAT was included in the Pulmonary–Symptomatic domain to complement objective measures of airflow limitation and exacerbation history by capturing the patient-centred dimension of disease severity. In the DWI, CAT was assigned a provisional weight of 0.12, corresponding to its tier in Table 1, while acknowledging that symptom burden represents one component of risk accumulation and may be influenced by factors beyond pulmonary impairment alone.

### 2.4. NT-proBNP (N-Terminal Pro–B-Type Natriuretic Peptide)

NT-proBNP was included in the risk stratification chart as a biochemical marker of cardiopulmonary stress and systemic burden. Its increase in COPD often reflects right ventricular pressure overload due to chronic hypoxic pulmonary vasoconstriction, left ventricular dysfunction, and systemic inflammation, which contribute to worse clinical outcome [41]. In a meta-analysis, NT-proBNP concentrations were higher during exacerbations than in stable COPD, in non-survivors than in survivors, and in patients with pulmonary hypertension or heart failure than in those without [42].

Several cohort and hospital-based studies have demonstrated that NT-proBNP is an independent risk factor for mortality in COPD [43,44]. During acute exacerbations, plasma NT-proBNP levels can rise and correlate with PaCO_2_, hypoxemia, and the risk of hospitalisation and ventilatory support. In a meta-analysis of seven studies, NT-proBNP above the study-specific cut-off predicted all-cause mortality both in patients with an exacerbation and in patients without exacerbation [44].

For these reasons, NT-proBNP was integrated within the Cardio–Systemic domain, representing the cardiovascular component of systemic involvement in COPD [20]. A provisional weight of 0.16 was assigned, the highest of any parameter in the index, because it is the only item for which the pooled adjusted effect estimate available in the literature exceeds 2.0 (Section 2.12); this reflects the ranking of the available estimates, not an established relative contribution.

### 2.5. C-Reactive Protein (CRP)

CRP was included in the risk stratification model as a systemic inflammatory biomarker reflecting the extrapulmonary component of COPD [20,34,45]. Unlike FEV_1_, which represents the main indicator of airflow limitation, CRP captures the underlying systemic inflammation, and elevated CRP predicts hospitalisation and death due to COPD [46]. Increased CRP concentrations have been associated with mortality in COPD in a meta-analysis [47], supporting its role as a marker of systemic inflammatory burden.

During acute exacerbations, CRP concentrations rise above stable-state values, in relation to the intensity of systemic inflammation and the presence of infection. In the ECLIPSE cohort, persistent systemic inflammation, defined as at least two of four biomarkers (white-cell count, CRP, IL-6 and fibrinogen) persistently in the upper quartile, was associated with higher all-cause mortality and exacerbation frequency than in patients without systemic inflammation [45], and in the Copenhagen City Heart Study a baseline CRP above 3 mg/L independently predicted hospitalisation and death due to COPD [46]. These findings suggest that CRP represents a potentially modifiable systemic risk signal that bridges pulmonary inflammation and outcomes.

Within the DWI, CRP was assigned a provisional weight of 0.12 under the Cardio–Systemic domain, corresponding to its tier in Table 1.

### 2.6. NYHA Functional Class

The New York Heart Association (NYHA) functional classification was included in this risk model as a composite indicator of global cardiovascular involvement in COPD. The NYHA classification allows us to integrate the effects of pulmonary and cardiovascular dysfunction, as well as reduced exercise tolerance. Although it was designed to evaluate heart failure, the NYHA class is a simple powerful descriptor of physical impairment. It correlates with respiratory dysfunction, dyspnoea, and exercise tolerance, which are symptoms related to COPD progression [48,49].

In patients with COPD, a higher NYHA class (III–IV) often reflects overlapping cardiac dysfunction, pulmonary hypertension, or systemic deconditioning, which contribute to worse outcomes [50,51,52]. Furthermore, comorbid heart failure is associated with higher hospitalisation and mortality in COPD [50,52], although estimates by NYHA class are not available (Section 2.12). The classification can predict long-term outcomes. It is also a marker to identify patients with combined cardiac and respiratory failure, which is usually considered a population characterised by high rates of hospitalisation and impaired quality of life.

Within the DWI, NYHA functional class was included in the Cardio–Systemic domain, together with NT-proBNP, since it expresses cardiac and cardiopulmonary functional limitation rather than metabolic status; this placement groups the two cardiac items so that their partly overlapping information is interpreted at domain rather than at item level (Section 2.12). A provisional weight of 0.12 was assigned to this parameter. Because no adjusted estimate by NYHA class is available in COPD, this weight rests on a proxy, the pooled estimate for comorbid heart failure (Table 1), and is among the least secure elements of the scheme; functional impairment is, moreover, a downstream expression of multiple interacting mechanisms.

### 2.7. Glycated Haemoglobin (HbA1c)

HbA1c was added to the multidimensional risk model as a marker of metabolic dysfunction and systemic oxidative stress conditions; dysglycaemia has been associated with lower lung function in general-population studies and with the risk of developing COPD [53,54,55]. Hyperglycaemia is recognised as a relevant comorbidity in COPD, even in the absence of overt diabetes [55]. Chronic hyperglycaemia promotes non-enzymatic protein glycation and systemic inflammation, resulting in endothelial dysfunction, impaired immune system responses, increased susceptibility to infection, and a decline in lung function. In COPD patients without diabetes, higher HbA1c has been associated with exacerbation status [56], and the stress hyperglycaemia ratio has been associated with in-hospital and intensive-care mortality in critically ill patients with COPD [57]. Poor glycaemic control also impairs respiratory muscle performance, exercise tolerance.

Within the DWI, HbA1c was assigned a provisional weight of 0.03 under the Metabolic–Nutritional domain, the lowest tier of the weighting scheme, because no adjusted effect estimate comparable to the categories used by the index could be identified in the literature (Section 2.12).

### 2.8. Body Mass Index (BMI)

BMI was included in the multidimensional risk stratification model as a surrogate marker of nutritional and metabolic status, both of which have a real influence on outcomes in COPD [25]. Low BMI reflects skeletal muscle wasting, systemic inflammation, and energy imbalance, which is associated with poor exercise tolerance, frequent exacerbations, and mortality. Conversely, high BMI values identify an “obese COPD” phenotype that is often complicated by obstructive sleep apnoea, systemic inflammation, and higher cardiovascular risk [58].

The relationship between BMI and mortality in COPD is non-linear: underweight is associated with higher mortality, whereas overweight and obesity are associated with lower mortality, the so-called obesity paradox [58,59]; in a meta-analysis, the protective association was lost in class III obesity, without a significant increase in risk, and in dose–response analysis it was no longer evident above about 35 kg/m^2^ [58]. We underline the protective role of adequate body mass in preserving respiratory muscle function and buffering catabolic stress during exacerbations. BMI is one of the four components of the BODE index, in which it contributed to the prediction of survival together with FEV_1_, dyspnoea and exercise capacity [25]. Furthermore, low BMI has been linked to frequent hospitalisations and reduced recovery after exacerbations, emphasising its dual role as both a marker and mediator of systemic vulnerability. For clinical users it should be noted that, in the DWI scoring rubric (Table 2), overweight and mild obesity (BMI ≥ 25 kg/m^2^) are deliberately assigned the lowest-risk score, in keeping with the obesity paradox literature, in which such patients show the most favourable survival; the simplified three-level ordinal metric does not distinguish class-III (morbid) obesity (>40 kg/m^2^), in which the protective association is lost [58], and this is acknowledged as a limitation (Section 4.6).

Within the DWI, BMI was placed under the Metabolic–Nutritional domain and assigned a provisional weight of 0.12.

### 2.9. Age

Age was introduced into the multidimensional risk stratification model, representing a punctual marker of physiological reserve, systemic vulnerability, and cumulative exposure to environmental and biological stressors. Aging contributes to the pathogenesis and prognosis of COPD through multiple anatomical, functional, and immunological mechanisms. Progressive loss of elastic lung recoil, reduction in respiratory muscle strength, endothelial dysfunction, and impaired immune response are often key mechanisms associated with ageing [3]. Patients with COPD frequently have cardiovascular comorbidity [60], and elderly subjects are often affected by different cardiovascular, metabolic, or musculoskeletal disorders that synergistically interact with airway inflammation, worsening patients’ outcomes.

Several longitudinal studies have demonstrated that advanced age is an independent predictor of exacerbation, hospitalisation, and mortality in COPD. Age is one of the three components of the ADO index (Age, Dyspnoea, Obstruction) [27], and in the CODEX index (Comorbidity, Obstruction, Dyspnoea, Exacerbation) it enters through the age-adjusted Charlson comorbidity index [29]. These findings highlight age as an important factor that increases the cumulative burden of disease, influencing treatment responses and prognosis.

Within the DWI, age was included under the Demographic–Exposure domain and assigned a provisional weight of 0.08. No estimate of the isolated effect of age categories was available: the tier was assigned from estimates obtained for composite indices, in which age is one of several components and its effect cannot be separated from theirs (Table 1). This weight therefore reflects the consistent presence of age in established indices rather than a measured independent contribution.

### 2.10. Smoking History

Smoking history was included in the multidimensional risk model as a core determinant of both disease initiation and progression, representing cumulative exposure to oxidative, inflammatory, and proteolytic stress. Tobacco smoke is the main environmental risk factor for COPD, inducing structural airway remodelling, parenchymal destruction, and persistent activation of innate and adaptive immune pathways [3,61,62]. The consequent inflammation and endothelial dysfunction extend beyond the lungs, predisposing individuals to cardiovascular and metabolic dysfunction; in cohort studies, smokers had an increased risk of exacerbation and faster loss of lung function [63,64].

While smoking cessation remains the most important intervention to slow disease progression, the effect of cumulative exposure continues to influence prognosis. Compared with never-smokers, ever-smokers have a higher risk of exacerbation and a faster decline in lung function [64], and in COPD the burden of multimorbidity increases with disease progression and age [65].

In this model, smoking history was incorporated into the Demographic–Exposure domain and assigned a provisional weight of 0.03.

### 2.11. Sex (Biological Sex)

Sex was considered for inclusion in the multidimensional model as a demographic and biological modifier of COPD presentation and clinical outcomes, including healthcare-use trajectories before diagnosis [66,67]. COPD was considered a predominantly male disease due to higher smoking habits; however, the burden of COPD among women has increased over the past two decades, reflecting changes in smoking habits and sex-specific susceptibility [66]. This change reflects both increasing exposure and sex-specific biological differences in airway structure, hormonal regulation, and inflammatory response.

Women with COPD tend to exhibit greater airway hyperresponsiveness, smaller airway calibre, higher symptoms, and decline in quality of life compared with men. In contrast, men are more likely to present with emphysema-predominant disease and cardiovascular comorbidities. Women with COPD have been reported to experience more exacerbations than men [66,68].

Nevertheless, while sex influences COPD phenotype and disease perception, the direction and independence of its association with mortality vary across cohorts; in a pooled analysis of Spanish cohorts, for example, male sex remained associated with higher mortality after adjustment [68]. Because of this heterogeneity, and because a fixed binary characteristic cannot occupy the full 0–1–2 ordinal range used for the other items without making the theoretical maximum of the index attainable in only one sex, biological sex has not been retained as a scored, weighted component of the Disease Weighted Index. It is instead treated as a pre-specified stratification covariate and candidate effect modifier, to be examined at the derivation stage for any independent prognostic contribution and for interaction with the remaining parameters. This choice removes the structural bias inherent in scoring an immutable characteristic, while preserving the information that sex conveys about phenotypic heterogeneity in COPD.

### 2.12. Development of the Disease Weighted Index (DWI)

Variables were selected a priori based on evidence of an association with prognosis in large observational cohorts, meta-analyses, and international guidelines. The final model incorporated ten parameters, grouped into four predefined domains representing complementary dimensions of COPD severity.

As this work is methodological and hypothesis-generating, it does not derive from the statistical analysis of a primary patient dataset, and it does not present coefficient estimates, ROC/AUC analyses or calibration measures obtained in a derivation cohort. The weights were nevertheless not assigned by unstructured judgement, although they remain expert-informed: they were obtained through an explicit ordinal procedure, in which every parameter was first assigned to a tier of prognostic magnitude on the basis of the effect estimates actually reported in the literature, and each tier was then mapped onto a fixed weight. Table 1 reports, for every parameter, the effect estimate that determined its tier, the source of that estimate and the resulting weight, so that each assignment can be reproduced or contested individually. The resulting weights are provisional, expert-informed specifications of the index. They are not estimates of the relative prognostic contribution of the parameters, which can be established only by joint estimation in patient-level data (Section 3.5).

**Step 1—Retrieval of the available effect estimates.** For each of the ten parameters we sought, in the studies cited in Section 2.1, Section 2.2, Section 2.3, Section 2.4, Section 2.5, Section 2.6, Section 2.7, Section 2.8, Section 2.9, Section 2.10 and Section 2.11, an adjusted effect estimate comparing the highest-risk with the reference category of that parameter, for mortality, hospitalisation or severe exacerbation. Preference was given, in this order, to meta-analyses, to large prospective cohorts and to single-centre studies. Where no such estimate exists, this is stated explicitly in Table 1. A proxy was used for one parameter only, NYHA functional class, for which no COPD-specific adjusted estimate by NYHA category could be identified; the pooled estimate for comorbid heart failure was adopted as the closest available proxy and is labelled as such in Table 1.

**Step 2—Allocation to tiers of prognostic magnitude.** Parameters were then allocated to four tiers by the authors’ judgement, guided by approximate bands of effect size rather than by deterministic rules: Tier 1, estimates of about 2.0 or more; Tier 2, estimates of about 1.5 to 2.0, or—for FEV_1_—status as a component of four of the five established multidimensional indices considered here (BODE, ADO, DOSE and CODEX; it is absent from COTE) [25,27,28,29], although as a single variable it discriminated less well than the composite BODE index in the BODE validation cohort (c  =  0.65 versus 0.74) [25]; Tier 3, estimates of about 1.2 to 1.5, the estimate closest to the 12-month prediction horizon being considered where several were available; Tier 4, no adjusted estimate comparable to the categories used by the index, the parameter being retained on mechanistic and clinical grounds pending empirical derivation. Because the available estimates differ in comparison, outcome and adjustment, these bands guided rather than determined the allocation, and for four parameters the allocation is not uniquely determined by them. For BMI, the single-cohort estimate (1.57) was preferred to the pooled estimate (1.39–1.42), which would indicate Tier 3, because its cut point (21 kg/m^2^) is the one adopted by the index; this departs from the order of preference stated in Step 1. For CAT, Tier 2 rests on the highest band of the source study (RR 1.50 for CAT 30–40), whereas the 20–29 band, which contains most of the highest DWI category, would indicate Tier 3. For smoking history, an adjusted estimate for current versus never smokers exists (adjusted OR 2.37 for subsequent exacerbation) [64], but the item was retained in the lowest tier because that estimate derives from a single cohort, refers to exacerbations only and does not address the grading of former smokers by pack years; single-cohort evidence is thus treated differently for BMI and for smoking history, which underlines that these allocations are judgements. For HbA1c, an adjusted estimate in patients with COPD exists (HR 1.28, 1.11–1.48, for all-cause death in the highest versus a middle HbA1c decile) [55], although its categories do not match those of the index. These four allocations are the most contestable of the scheme; under a strict application of the bands, BMI, CAT and HbA1c would be in Tier 3 and smoking history in Tier 1, and all four are pre-specified for re-estimation (Section 3.5).

**Step 3—Mapping of tiers onto weights.** The four tiers were mapped onto the fixed weights 0.16, 0.12, 0.08 and 0.03 respectively. The scale was chosen so that the weights sum to 0.98 given the observed distribution of parameters across tiers, and so that the ratio between the highest and the lowest weight is approximately 5:1; this ratio is a judgement that cannot be checked against the data, since Tier 4 parameters have no comparable estimate. No parameter-specific adjustment was applied after tier allocation. The weighting scheme presented here is treated as a fixed a priori specification: no further manual adjustment of the weights, or of the set of parameters, will be made. Any subsequent change will occur solely through the pre-specified, data-driven re-estimation of the derivation phase (Section 3.5), in which the weights will be obtained from formally pooled or, where available, meta-analytic estimates and ultimately from multivariable modelling in the derivation cohort, rather than from the single studies used to order the tiers at this stage.

**Table 1:** Allocation of the ten parameters of the Disease Weighted Index (DWI) to tiers of prognostic magnitude, and the resulting weights. For each parameter the table reports the adjusted effect estimate that determined the tier, together with its source. Where no estimate comparable to the categories used by the index could be identified, this is stated; the single proxy estimate used (heart-failure status for NYHA class) is identified as such. The estimates derive from studies differing in population, outcome definition and adjustment set and were not pooled by formal meta-analysis; the resulting weights are therefore provisional, expert-informed specifications, reproducible but not established prognostic contributions or empirically estimated regression coefficients, and are intended to be re-estimated in a derivation cohort (Section 3.5).

Parameter	Adjusted Effect Estimate Used for Tier Allocation	Source	Tier	Weight
NT-proBNP	Pooled HR for all-cause mortality with NT-proBNP above the study-specific cut-off: 2.87 (1.70–4.84) in patients with exacerbation and 3.34 (1.04–10.77) in patients without exacerbation; overall 2.80. Meta-analysis of seven studies; cut-offs varied across studies	[44]	1	0.16
FEV_1_ (% predicted)	No adjusted HR reported for FEV_1_ categories in isolation. Allocated on convergent evidence: component of BODE, ADO, DOSE and CODEX (not COTE); c = 0.65 as a single variable, versus 0.74 for the BODE index, in the BODE validation cohort	[25,27,28,29]	2	0.12
CAT score	Adjusted RR 1.50 for CAT 30–40 and 1.37 for CAT 20–29, versus CAT 0–9, for exacerbation within six months; the 20–29 band, which contains most of the highest DWI category, would indicate Tier 3 (Step 2)	[39]	2	0.12
CRP	Pooled HR 1.61 (95% CI 1.12–2.30) in studies using a 3 mg·L^−1^ threshold; 1.53 (1.32–1.77) overall, both for late mortality. Cohort HR 1.4 for COPD hospitalisation and 2.2 for COPD death, CRP > 3 versus ≤ 3 mg·L^−1^	[46,47]	2	0.12
NYHA class	No adjusted HR for NYHA class in COPD. Pooled HR 1.61 (1.38–1.83) for all-cause mortality in comorbid heart failure versus no heart failure, used as the closest available proxy (not an estimate for NYHA class; studies with spirometry-confirmed COPD; publication bias reported)	[50]	2	0.12
BMI	RR 1.57 (1.24–1.99) for BMI ≤ 21 versus 21–25 kg·m^−2^, adjusted for age and FEV_1_; pooled RR 1.39 (1.28–1.50) for underweight versus normal BMI, 1.42 when adjusted for age, sex, smoking and FEV_1_. The single-cohort estimate was used; the pooled estimate would indicate Tier 3 (Step 2)	[58,59]	2	0.12
Exacerbations (12 months)	Adjusted HR 1.35 for ≥ 2 versus 1 event in the preceding year, for time to first exacerbation	[39]	3	0.08
Age	No isolated HR for age categories. CODEX index, which incorporates age through the age-adjusted Charlson index: HR 1.5 (1.2–1.8) for mortality at three months and 1.3 (1.2–1.5) at one year; the one-year estimate, closest to the 12-month prediction horizon, was used for tier allocation. These estimates refer to the composite index score rather than to a category contrast for age, and are therefore not on the same scale as the estimates for the other parameters	[29]	3	0.08
HbA1c	No adjusted estimate comparable to the categories used by the index. In UK Biobank participants with COPD, HR 1.28 (1.11–1.48) for all-cause death, highest versus a middle HbA1c decile [55]; the cited cross-sectional COPD study excluded patients with diabetes. Allocation to Tier 4 is a judgement (Step 2)	[56]	4	0.03
Smoking history	Adjusted OR 2.37 for subsequent exacerbation in current versus never smokers (2.06 in former smokers), single cohort; no estimate by pack-years. Allocation to Tier 4 departs from the bands and is a judgement (Step 2)	[64]	4	0.03

Three consequences of this procedure are stated explicitly rather than left to the reader. First, five of the ten parameters—FEV_1_, NYHA class, age, HbA1c and smoking history—could not be anchored to an adjusted estimate matching the categories used by the index (for NYHA class a proxy was used, and for age only estimates for composite indices were available), the estimates used for CAT, exacerbation history and BMI match the categories of the index only partly, and two of them, HbA1c and smoking history, have been placed in the lowest tier by judgement (Step 2). Their weights are the least defensible element of the model and the first that empirical derivation should revise. Second, because the estimates were abstracted from studies differing in population, outcome definition and adjustment set, and were deliberately not pooled by formal meta-analysis, the procedure yields at most a provisional ordering of the parameters and does not establish their relative predictive contributions; this is why an ordinal mapping was preferred to an arithmetic transformation of individual hazard ratios, which would have conveyed a spurious impression of precision. The procedure therefore assumes only that the estimates can be ranked, not that estimates obtained in different populations, for different endpoints and with different adjustment sets are commensurable on a ratio scale, and its adequacy will be tested empirically against two alternative specifications: equal weights (the unweighted sum of the items) and weights re-estimated in the derivation cohort (Section 3.5). Third, several parameters are mutually correlated—NT-proBNP and NYHA class both reflect cardiac strain, and CRP and exacerbation history share an inflammatory pathway—so that the additive model may over-represent the corresponding constructs. NYHA class and NT-proBNP were placed in the same domain so that the domain sub-score, rather than the individual item, is the quantity to be interpreted, and joint estimation in a single multivariable model is foreseen at the derivation stage (Section 3.5).

A further caveat deserves emphasis because it bears on the premise of the index rather than on its execution. In the derivation of the BODE index, the authors reported that assigning different weights to the individual components produced no corresponding increase in predictive value [25]. That result was obtained in a four-component index built exclusively from pulmonary and functional variables, and it does not necessarily extend to a model that adds circulating biomarkers spanning distinct biological pathways; but it establishes that the benefit of weighting cannot be assumed, and that a weighted index must demonstrate superiority over the unweighted sum of the same items. This comparison is therefore pre-specified as a primary analysis of the derivation phase (Section 3.5).

Each parameter was categorised into three ordinal risk levels (0–1–2) using clinically meaningful cut-offs derived from international guidelines and large cohort studies (Table 2); the source and rationale of every threshold are detailed in Section 2.13 and Table 3. Higher scores indicate increasing disease burden or risk.

Parameter weights correspond to the tier of prognostic magnitude to which each parameter was allocated (Table 1); they are provisional specifications to be re-estimated in the derivation phase.

The following provisional normalised weights were applied:

Pulmonary–Symptomatic domain (total weight = 0.32):

- FEV_1_ (% predicted): 0.12;
- Exacerbations (previous 12 months): 0.08;
- CAT: 0.12.

Cardio–Systemic domain (total weight = 0.40):

- NT-proBNP: 0.16;
- CRP: 0.12;
- NYHA functional class: 0.12.

Metabolic–Nutritional domain (total weight = 0.15):

- HbA1c: 0.03;
- BMI: 0.12.

Demographic–Exposure domain (total weight = 0.11):

- Age: 0.08;
- Smoking history: 0.03.

The ten retained parameter weights sum to 0.98; the index is normalised by the maximum attainable weighted score (twice this sum, i.e., 1.96), preserving a 0–100 range together with internal consistency and interpretability. Domain weights were calculated as the sum of the weights of their constituent parameters, and should be read, like the parameter weights, as provisional specifications rather than as the relative contribution of each disease dimension to overall risk.

**Table 2:** Scoring rubric of the Disease Weighted Index (DWI). Each parameter was categorised into three ordinal risk levels (0–1–2) using clinically meaningful thresholds derived from international guidelines and large observational studies; the last column reports the normalised weight assigned to each parameter. CAT cut-offs were selected according to validated categories of symptom burden. The source and rationale of every threshold are reported in Table 3.

Parameter	Domain	Score = 0 (Low)	Score = 1 (Moderate)	Score = 2 (High)	Weight
FEV_1_ (% predicted)	Pulmonary–Symptomatic	≥80% (GOLD 1)	50–79% (GOLD 2)	<50% (GOLD 3–4)	0.12
Exacerbations (last 12 months)	Pulmonary–Symptomatic	0	1 (moderate)	≥2 or ≥1 hospitalisation	0.08
CAT score	Pulmonary–Symptomatic	< 10	10–20	>20	0.12
NT-proBNP	Cardio–Systemic	<125 pg/mL (any age)	125–450 pg/mL if age < 75 y; 125–900 pg/mL if age ≥ 75 y	>450 pg/mL if age < 75 y; >900 pg/mL if age ≥ 75 y	0.16
CRP	Cardio–Systemic	<3 mg/L	3–5 mg/L	>5 mg/L	0.12
NYHA class	Cardio–Systemic	I	II	III–IV	0.12
HbA1c	Metabolic–Nutritional	<6.5%	6.5–7.5%	>7.5%	0.03
BMI (kg/m^2^)	Metabolic–Nutritional	≥25	21–24.9	<21	0.12
Age (years)	Demographic–Exposure	<65	65–75	>75	0.08
Smoking history	Demographic–Exposure	Never smoker, or former smoker ≤ 10 pack-years	Former smoker > 10 pack-years	Current smoker	0.03

For each patient, a parameter-specific score *s_i_* ∈ {0, 1, 2} was assigned. A global weighted score was then calculated as

$$\mathrm{Weighted}\;\mathrm{Sum}=\sum_{i=1}^{10}w_{i}\cdot s_{i}$$

Given that the maximum attainable weighted sum is 1.96 (twice the sum of the ten retained weights, whose total is 0.98), the score was normalised to a 0–100 scale:

$$\mathrm{Disease}\;\mathrm{Weighted}\;\mathrm{Index}\;\left(\mathrm{DWI}\right)=\left(\frac{\sum_{i=1}^{10}w_{i}\cdot s_{i}}{1.96}\right)\times 100$$

where

- *w_i_* represents the normalised weight assigned to the *i*-th parameter;
- *s_i_* represents the parameter-specific risk score (0, 1, or 2);
- the denominator, equal to 1.96 (twice the sum of the ten retained weights), corresponds to the maximum attainable weighted sum, given that Σ*w_i_* = 0.98 and *s_i_* ≤ 2.

This normalisation yields a continuous score ranging from 0 to 100, with higher values indicating progressively greater multidimensional disease burden and risk of adverse outcomes (Table 2).

### 2.13. Selection and Justification of the Cut-Off Values

Cut-off values were not selected ad hoc. For each parameter, thresholds were taken, in decreasing order of priority, from (i) the categories defined by international guidelines or by position papers of the relevant scientific societies, (ii) the categories used in the studies from which the corresponding effect size was abstracted in Table 1, and (iii), when neither was available, the thresholds most widely adopted in the COPD literature. The source and rationale of every threshold are reported in Table 3. The thresholds that required a specific methodological choice are discussed below.

**FEV_1_.** The cut-points of the BODE and CODEX indices (65%, 50% and 35% of predicted) do not correspond to any staging system in current clinical use, and the 65% threshold in particular has no counterpart in the GOLD classification. The GOLD spirometric grades were therefore adopted: score 0 = FEV_1_ ≥ 80% (GOLD 1), score 1 = 50–79% (GOLD 2), score 2 < 50% (GOLD 3–4). This preserves the three-level ordinal structure of the index while making each DWI item directly interpretable against the classification clinicians already use. Because the categorisation of airflow limitation depends on the reference equation, the standard is specified as follows: FEV_1_ is to be measured after bronchodilator administration, as required by GOLD, and percent predicted is to be calculated with the Global Lung Function Initiative (GLI) equations recommended by the current ERS/ATS interpretive standard [69], in their race-neutral GLI Global (2022) version [70]. Where a centre uses a different, locally validated reference set, the equation must be recorded. In the derivation phase, agreement between the categories obtained with GLI Global and with the GLI-2012 ethnic-specific equations will be quantified, and FEV_1_ z-scores will be examined as an alternative continuous parametrisation (Section 3.5).

**NT-proBNP.** Natriuretic peptide concentrations rise physiologically with age and with declining renal function. Applying a single upper threshold therefore systematically over-scores older patients, which is particularly undesirable in an index in which age is also scored separately. Age-adjusted thresholds have consequently been adopted: 125 and 450 pg/mL for patients aged < 75 years, and 125 and 900 pg/mL for patients aged ≥ 75 years. The lower threshold of 125 pg/mL is retained unchanged for all ages, since it is the age-independent rule-out threshold for chronic heart failure [71]. The upper thresholds were adapted from the age-stratified rule-in values for acute heart failure (450, 900 and 1800 pg/mL at <50, 50–75 and >75 years) [71] and simplified to two strata for use in stable outpatients; because those values were derived in patients with acute dyspnoea and have not been validated in stable COPD, they are a pragmatic choice that the derivation phase will recalibrate. This adjustment attenuates, but does not eliminate, the entanglement between the NT-proBNP and Age items: because the NT-proBNP thresholds are themselves age-stratified, age influences the index in two ways—as a separately scored item and as the variable that sets the NT-proBNP cut-points—so that the two items are not fully independent. Age is nonetheless retained, because it captures aspects of physiological reserve and cumulative risk that extend beyond cardiac stretch alone; this residual overlap is pre-specified for formal evaluation by joint estimation in a single multivariable model at the derivation stage (Section 3.5), where age may be re-weighted or, if redundant, removed.

**Smoking history.** The three levels were defined so as to be mutually exclusive and jointly exhaustive, with former smokers with ≤10 pack-years explicitly assigned to the lowest category: score 0 = never smoker, or former smoker with ≤10 pack-years; score 1 = former smoker with >10 pack-years; score 2 = current smoker, irrespective of cumulative exposure.

**CAT.** The CAT is conventionally reported on a four-tier scale of low, medium, high and very high impact. It was deliberately collapsed into three levels (<10, 10–20, >20) so that all ten parameters share the same 0–1–2 ordinal metric, which is a structural requirement of the weighted sum; 10 is the symptom threshold used by GOLD to separate the less and more symptomatic groups, and 20 identifies high symptom impact in the CAT user guide. The cost of this simplification is the loss of the distinction between the high and very high impact bands, which is acknowledged as a limitation and which could be recovered, at the price of a heterogeneous metric, by adopting a 0–3 scale for this item alone at the derivation stage.

**Sex.** Biological sex is not part of the weighted scoring of the index and is retained only as a pre-specified stratification covariate (Section 2.11); it therefore does not appear in the scoring rubric (Table 2) or in the weighting scheme.

**Table 3:** Source and rationale of the cut-off values used in the Disease Weighted Index (DWI). GOLD, Global Initiative for Chronic Obstructive Lung Disease; HFA-ESC, Heart Failure Association of the European Society of Cardiology.

Parameter	Thresholds (Score 0/1/2)	Source and Rationale
FEV_1_ (% predicted)	≥80/50–79/<50	Post-bronchodilator FEV_1_, % predicted by the GLI Global (2022) equations [69,70]. GOLD spirometric grades 1–4, collapsed into three levels by merging GOLD 3 and 4 to preserve a common three-level metric [3,72]; this discards the distinction retained by the BODE index at FEV_1_ ≤ 35%. The alternative cut-points of the BODE and CODEX indices (≥65%, 50–64%, 36–49%, ≤35%) [25,29] are evidence-based but do not correspond to any staging system in current use.
Exacerbations	0/1 moderate/≥2 or ≥1 hospitalisation	GOLD definition of the high-risk exacerbator [3,7,35]. The effect size in Table 1 derives from a study of patients with at least one previous exacerbation that compared ≥2 with 1 event [39]; no estimate for the 0 category was available.
CAT score	<10/10–20/>20	10 is the GOLD symptom threshold [3]; 20 separates medium from high impact in the impact bands of the CAT user guide. Four-tier scale collapsed to three to preserve a common ordinal metric; CAT development and measurement properties [36,37].
NT-proBNP	<125/125–450 (<75 y) or 125–900 (≥75 y)/>450 (<75 y) or >900 (≥75 y) pg/mL	The age-independent rule-out threshold for chronic heart failure is 125 pg/mL; upper thresholds adapted from the age-stratified rule-in values for acute heart failure (450, 900 and 1800 pg/mL at <50, 50–75 and >75 years) and simplified to two age strata to avoid systematic over-scoring of older patients; not validated in stable COPD and pre-specified for recalibration [71].
CRP	<3/3–5/>5 mg/L	The threshold used in the cohort and meta-analysis from which the effect size derives is 3 mg/L [46,47]; 5 mg/L is a pragmatic upper cut-off, corresponding to the upper reference limit of standard assays, without outcome-based validation in COPD; pre-specified for recalibration.
NYHA class	I/II/III–IV	Native categories of the classification [48,50]; III and IV merged to preserve a common three-level metric.
HbA1c	<6.5/6.5–7.5/>7.5%	The diagnostic threshold for diabetes is 6.5%; 7.5% is a pragmatic cut-off within the range of the less stringent glycaemic targets commonly adopted for older patients with comorbidity. These categories were not used in the cited COPD studies [55,56] and are pre-specified for recalibration.
BMI	≥25/21–24.9/<21 kg/m^2^	The threshold used in the BODE index is 21 kg/m^2^, and in the BODE-cohort analysis reporting an adjusted risk ratio of 1.57 for values below it. Severe obesity is not scored as high-risk: overweight and obesity are associated with reduced mortality, and the protective association is lost in class III obesity without a significant increase in risk [25,58,59].
Age (years)	<65/65–75/>75	Conventional geriatric cut points (65 and 75 years). They do not reproduce the ADO index, which scores age in bands [27], or the CODEX index, which incorporates age through the age-adjusted Charlson index [29]; continuous modelling of age is pre-specified (Section 3.5).
Smoking history	Never or former ≤ 10 pack-years/former > 10 pack-years/current	Categories defined to be mutually exclusive and jointly exhaustive; current smoking is scored highest irrespective of cumulative exposure, given its association with exacerbation risk and faster decline in lung function [63,64]; 10 pack-years is a conventional cut-off separating minimal from substantial cumulative exposure in former smokers, without outcome-based validation in COPD.

### 2.14. Scoring When Parameters Are Unavailable

In routine practice, not all ten parameters are available at every encounter: NT-proBNP and HbA1c in particular are not measured universally in stable COPD outpatients. Rather than rendering the index non-computable, the DWI is renormalised over the parameters actually available. Denoting by A the set of available parameters:

DWI* = [Σ(i ∈ A) w_i_ · s_i_]/[2 · Σ(i ∈ A) w_i_] × 100

This preserves both the 0–100 range and the relative contribution of the observed items, and is equivalent to assuming that the missing parameters carry the average risk level of those observed. Two safeguards apply. First, the index should not be computed when the weights of the available parameters sum to less than 0.70, nor when an entire domain is missing, since in either case the multidimensional nature of the estimate is lost. Second, whenever renormalisation is applied the result should be reported as DWI*, accompanied by the list of missing items, because renormalised and complete-case values are not strictly interchangeable: a DWI* of 50 computed without NT-proBNP conveys less information than a complete DWI of 50, and the two should not be compared within the same patient over time.

Single imputation of missing biomarkers is explicitly discouraged in the absence of a derivation dataset, since it would introduce an unquantifiable bias into an index that has not yet been calibrated. In the derivation phase the mechanism and the impact of missingness will be handled formally through multiple imputation, and the discrimination of the complete and of the reduced versions of the index will be compared directly, so that the minimum data set required to compute a clinically meaningful DWI can be defined empirically rather than by convention.

### 2.15. Worked Examples

To make the computation explicit and reproducible, three hypothetical patients are scored through the formula in Table 4. These are illustrative constructions and not real patients; no clinical data were used.

Patient 1 is a 62-year-old woman, a former smoker with 15 pack-years, with FEV_1_ 62% of predicted, one moderate exacerbation in the preceding 12 months, CAT 14, NT-proBNP 90 pg/mL, CRP 2.1 mg/L, NYHA class I, HbA1c 5.9% and BMI 26.4 kg/m^2^. Patient 2 is a 71-year-old man, a former smoker with 30 pack-years, with FEV_1_ 68% of predicted, no exacerbations in the preceding 12 months, CAT 9, NT-proBNP 620 pg/mL, CRP 6.2 mg/L, NYHA class II, HbA1c 7.8% and BMI 32.0 kg/m^2^. Patient 3 is a 78-year-old man, a current smoker with 45 pack-years, with FEV_1_ 41% of predicted, two exacerbations of which one required hospitalisation, CAT 24, NT-proBNP 1250 pg/mL, CRP 4.2 mg/L, NYHA class III, HbA1c 7.9% and BMI 22.4 kg/m^2^.

Patient 2 illustrates the specific gap that the index is intended to fill, and also the reason why the domain sub-scores should be reported alongside the total. With GOLD 2 spirometry, no exacerbations in the preceding year and a CAT score below 10, this patient would be assigned to GOLD group A and would receive a low score on any index built exclusively from pulmonary and functional variables. His total DWI of 49.5 places him at the upper end of the moderate band, but his Cardio–Systemic sub-score is 85% of the attainable maximum, driven by markedly elevated NT-proBNP and CRP together with NYHA class II, and it is that sub-score, rather than the total, that indicates where the risk lies and which intervention might be considered. Whether this reclassification identifies a genuinely higher event rate, or merely relabels patients without prognostic gain, is precisely the hypothesis that the validation programme described in Section 3.5 is designed to test. Patients 1 and 3 illustrate the two extremes of the scale.

## 3. Proposed Framework

### 3.1. Disease Weighted Index and Risk Stratification

**Figure 1:**
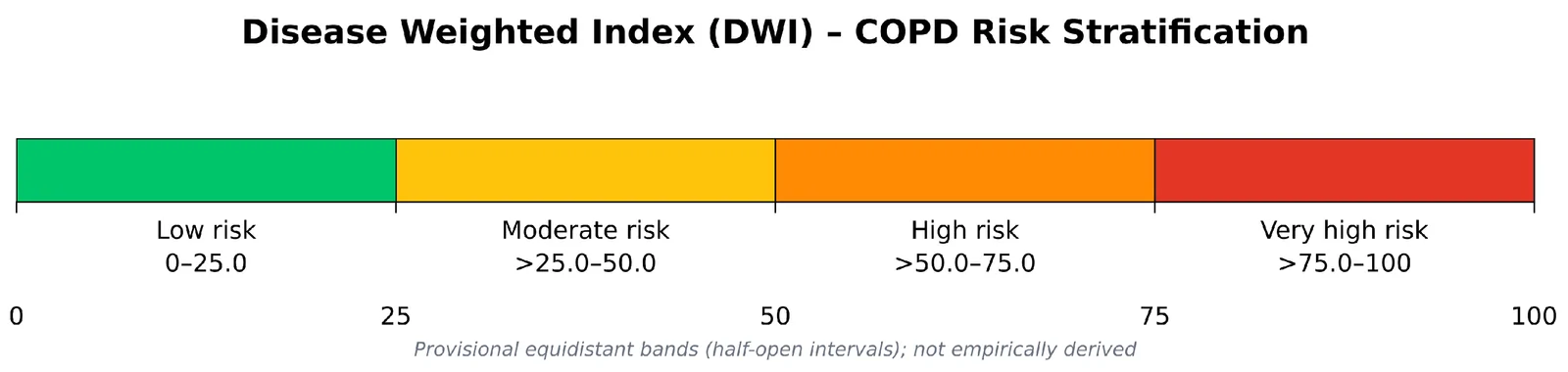
Colour-coded Disease Weighted Index (DWI) risk-stratification scale. The normalised DWI ranges from 0 to 100 and is divided into four categories: low (0–25.0), moderate (>25.0–50.0), high (>50.0–75.0) and very high (>75.0–100) risk, with increasing values intended to indicate progressively greater multidimensional disease burden. The boundaries are provisional and have not been derived empirically.

Based on the normalised DWI, patients would be stratified into four provisional risk categories, each linked to a proposed interpretation (Figure 1 and Table 5). Because the DWI is continuous, the categories are defined as contiguous half-open intervals (0–25.0, >25.0–50.0, >50.0–75.0 and >75.0–100), so that every attainable value, including fractional ones, belongs to exactly one category; the index is reported to one decimal place. The boundaries are equidistant divisions of the scale and have no empirical basis at this stage (Section 3.3).

### 3.2. Interpretation of DWI Values and of Their Changes over Time

Because the DWI is a weighted sum of ordinal items, its granularity is finite and can be stated exactly. The smallest possible non-zero change is approximately 1.5 points, produced by a one-level change in an item with the lowest weight (0.03); a one-level change in the most heavily weighted parameter, NT-proBNP, moves the index by 8 points, and a one-level change in any Tier 2 parameter by 6 points. A difference of 10 points therefore corresponds, approximately, to a one-level change in one Tier 2 and one Tier 3 parameter (6.1 + 4.1 points), or in NT-proBNP together with a Tier 4 parameter (8.2 + 1.5 points). Since the four risk bands are 25 points wide, a 10-point change does not necessarily produce reclassification, and two patients differing by 10 points may well fall in the same provisional category.

No minimal clinically important difference (MCID) can legitimately be claimed for the DWI at this stage. An MCID cannot be derived from the structure of a score: it requires anchor-based or distribution-based estimation in a cohort in which the score and a clinical outcome are measured concurrently, and no such cohort exists for the DWI. The crossing of a risk-band boundary is not used as an anchor: the boundaries are arbitrary at this stage, and treating the crossing of one as clinically meaningful would be circular until the thresholds and their clinical consequences have been established externally. The MCID will therefore be estimated only against anchors measured independently of the band structure—the established CAT MCID of 2 points [38], the occurrence of a moderate or severe exacerbation, and a global rating of change in health status—supported by a distribution-based estimate corresponding to half a standard deviation of the DWI distribution in the derivation cohort. Because the CAT is itself a component of the index, the CAT-anchored estimate will also be computed with the CAT item removed. Until these analyses have been performed, differences in DWI should be interpreted qualitatively and must not be used to define response to treatment.

### 3.3. Proposed Follow-Up Pathways (Hypotheses for Future Evaluation)

**Figure 2:**
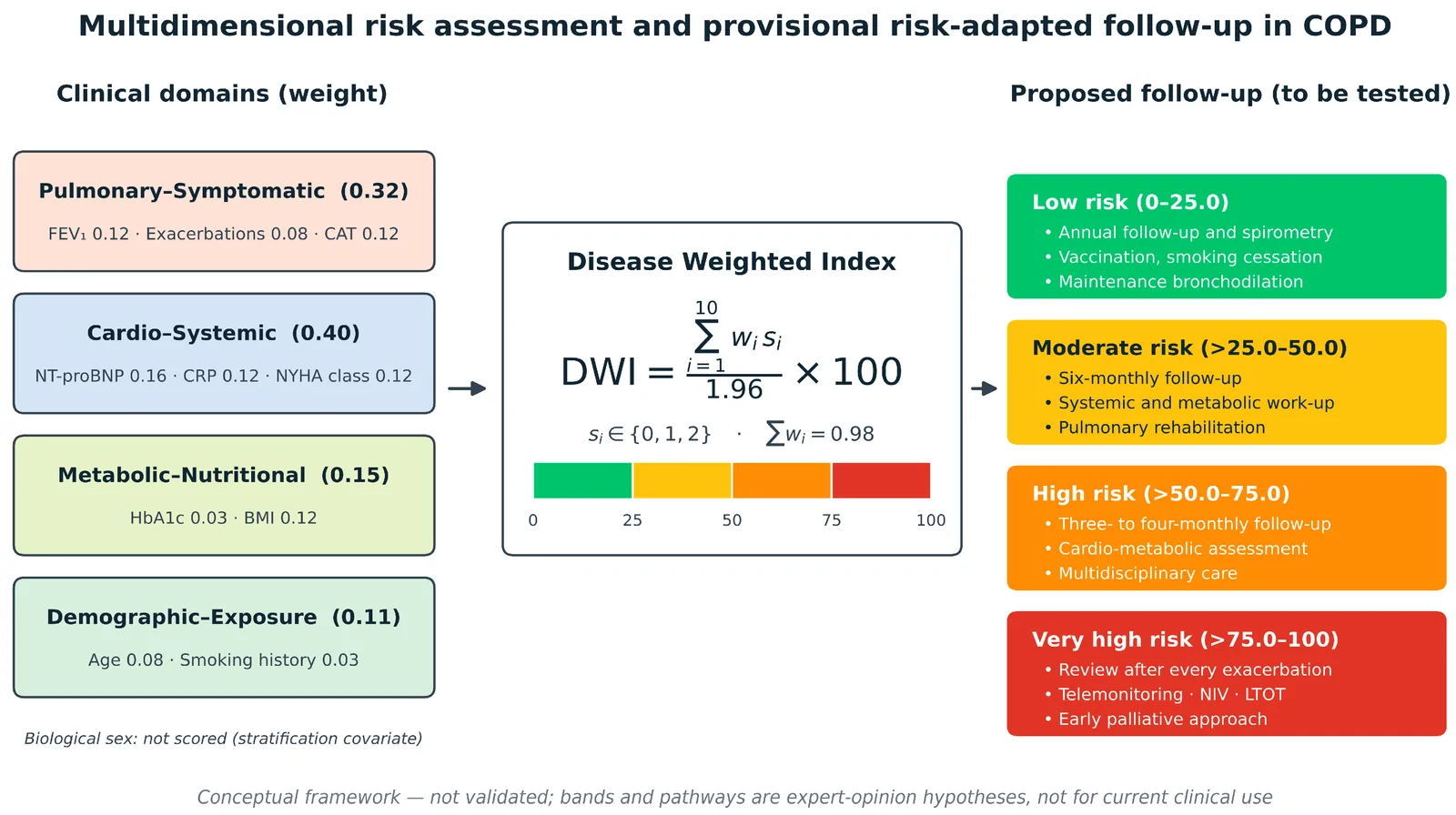
Proposed multidimensional COPD risk stratification and hypothesised risk-adapted follow-up based on the Disease Weighted Index (DWI). Clinical parameters are grouped into four domains (Pulmonary–Symptomatic, Cardio–Systemic, Metabolic–Nutritional and Demographic–Exposure) and combined into a weighted index ranging from 0 to 100. Patients are stratified into four risk categories (low, moderate, high and very high risk), each associated with an illustrative follow-up intensity proposed for prospective evaluation; these pathways reflect expert opinion and are not intended for current clinical use. The ten scored parameters carry weights summing to 0.98, and the weighted sum is normalised by its maximum (1.96). Biological sex is not scored and is shown only as a pre-specified stratification covariate.

For prospective evaluation, we propose a hypothetical risk-adapted follow-up scheme in which the intensity of follow-up and interventions would be tailored to the DWI category (Figure 2). It is critical to emphasise that these follow-up intervals and interventions represent hypotheses based on expert opinion and current clinical practices, rather than evidence-derived guideline consensus. They are intended as a practical baseline to be tested prospectively. The risk bands to which they are attached are themselves provisional: in the derivation phase they will be replaced by thresholds defined on the calibrated predicted 12-month risk of the primary outcome, and the clinical utility of those thresholds will be examined by decision curve analysis (Section 3.5). The pathways described below therefore indicate how the index could be operationalised in a prospective study; they are not recommendations for current practice, in which management should continue to follow GOLD and the other applicable guidelines.

In such a study, patients classified as low risk would be managed with annual clinical follow-up, yearly spirometry, reinforcement of smoking cessation strategies, vaccination, and optimisation of maintenance bronchodilator therapy, with a primary focus on prevention and lifestyle counselling [40]. Moderate-risk patients would receive closer monitoring, periodic functional assessment, targeted laboratory evaluation of systemic and metabolic markers, referral to pulmonary rehabilitation programs, and early optimisation of inhaled pharmacological treatment [40,73]. In high-risk patients, characterised by frequent exacerbations and/or relevant systemic involvement, intensified follow-up would be tested, together with comprehensive respiratory and cardiometabolic assessment, optimisation of pharmacological therapy including triple inhaled therapy when indicated, and a multidisciplinary approach involving pulmonologists, cardiologists, physiotherapists, and dietitians [73]. Finally, very-high-risk patients would receive close surveillance, particularly in the post-exacerbation period, advanced respiratory evaluation, cardiologic reassessment, consideration of telemonitoring strategies, evaluation for home non-invasive ventilation and long-term oxygen therapy, and early integration of palliative care approaches when appropriate [74,75,76]. The cited references describe the individual interventions in their own indications; for some, such as long-term oxygen in patients with moderate desaturation, the evidence is negative [76]. No benefit of allocating these interventions according to the DWI has been demonstrated, and none is claimed.

### 3.4. Conceptual Comparison with Established Multidimensional Indices

Because several validated multidimensional indices already exist, the DWI can only be justified if it addresses something they do not. Table 6 summarises the comparison. Two points require emphasis, and the first is unfavourable to the present proposal: every comparator listed has been derived and validated in patient cohorts, whereas the DWI has not, so that on the criterion of evidential status the established indices are at present unequivocally superior, and none of them should be replaced by the DWI on the basis of this work.

The second point concerns scope. BODE, ADO and DOSE summarise the pulmonary and functional dimension of the disease, and their components are predominantly respiratory and functional, with the addition of body mass index, age or smoking status. CODEX and COTE do incorporate comorbidity, but they do so through a comorbidity count or a weighted list of diagnoses rather than through measurable biological signals, and both were derived in specific settings—CODEX in patients discharged after an exacerbation, COTE for the estimation of mortality attributable to individual comorbid conditions. None of the five includes a circulating biomarker of cardiac strain, systemic inflammation or glycaemic control, and none was designed to be recomputed at each outpatient visit from parameters already present in the routine record. The DWI is proposed to occupy this specific space, and its incremental value must be judged, at the derivation stage, exactly on this ground: whether adding NT-proBNP, CRP and HbA1c to a BODE-like or CODEX-like backbone improves discrimination, reclassification and calibration. Should it not, the simpler and already validated indices are to be preferred, and this should be stated as clearly as the hypothesis itself.

### 3.5. From Concept to Evidence: A Pre-Specified Validation Roadmap

This study is methodological and hypothesis-generating. External validation in independent cohorts and prospective assessment are required to establish predictive performance, re-estimate the weights, and evaluate whether longitudinal changes in DWI track progression and treatment response.

Because the value of a conceptual proposal lies principally in its testability, the design of the test is specified here, so that it can be criticised, improved, or executed by others. The TRIPOD statement and its TRIPOD + AI update [77,78] are used here not as a reporting checklist for the present article, which reports no model development or validation, but as the framework against which the future derivation and validation studies will be planned and reported. Table 7 lists, for each component of the framework, what is specified conceptually in this work and what remains to be established empirically. We propose a three-phase programme, aligned with the TRIPOD reporting framework [77,78] and with the recommended steps for the development and validation of clinical prediction models [79].

**Prediction target and horizon.** The primary outcome is the first occurrence, within 12 months of each assessment, of any of the following: severe exacerbation, defined as an exacerbation requiring hospital admission or an emergency department visit; all-cause unplanned hospitalisation; or all-cause death. All-cause rather than COPD-related hospitalisation is specified because several components of the index target cardiovascular and metabolic risk, whose consequences a respiratory-specific definition would miss. Each component of the composite, and the same composite over 24 months, will be analysed as secondary outcomes.

**Phase 1—Retrospective derivation and re-estimation of the weights.** In a multicentre cohort of stable COPD outpatients with at least 24 months of follow-up and complete availability of the ten parameters, the weights will be re-estimated jointly in a single multivariable Cox model, with the primary outcome defined above. The literature-anchored weights presented here will serve as the pre-specified comparator, and the two versions of the index will be compared on discrimination and calibration. The sample size will be determined with the criteria proposed by Riley et al. for the development of clinical prediction models [80], which supersede the rule of ten events per candidate parameter; the latter (at least 100 events) is retained only as an absolute minimum. Estimated glomerular filtration rate and haemoglobin will be collected as candidate additional variables and tested for incremental value. Where available, six-minute walk distance, measured according to the ERS/ATS technical standard [81], will be collected as a candidate variable for an extended version of the index, together with the Timed Up and Go test and the Berg Balance Scale in patients aged 65 years or older; the incremental value of each over the ten core items will be tested formally (Section 4.4). Collinearity among the items, in particular between NT-proBNP and NYHA class, between NT-proBNP and age, and between CRP and exacerbation history, will be quantified with variance inflation factors and examined by joint estimation in the same multivariable model; a parsimonious specification in which the four domain sub-scores, rather than the individual items, enter the model will be compared with the item-level specification, and the simpler structure will be preferred if its performance is equivalent. The agreement of FEV_1_ categories across reference equations will be assessed as described in Section 2.13. Two comparisons are pre-specified as primary at this stage: the re-estimated weights against the literature-anchored weights proposed here, and the weighted index against the unweighted sum of the same ten items, the latter to establish whether weighting confers any benefit at all [25].

**Phase 2—Internal and external validation.** Internal validation by bootstrap resampling with correction for optimism, followed by geographical and temporal external validation in independent cohorts. The pre-specified comparative analysis is against the BODE and CODEX indices: the DWI will be considered to add value only if it improves the c-statistic and yields a positive net reclassification improvement over these indices while retaining adequate calibration in the external cohorts. The clinical utility of the index, and of the risk thresholds that will replace the provisional bands, will be assessed by decision curve analysis [82].

**Phase 3—Prospective and longitudinal evaluation.** Repeated measurement at fixed intervals in the stable state, in order to determine whether DWI trajectories predict outcomes beyond the baseline value, to estimate a minimal clinically important difference by anchor-based methods with anchors external to the band structure (Section 3.2) and by distribution-based methods, and to quantify the effect of concomitant therapy on the individual components. Only after this phase would an interventional evaluation of DWI-guided follow-up intensity be justified.

We regard it as important to state in advance what would count as a negative result. Should the biomarker components add nothing to a BODE- or CODEX-like backbone, or should the re-estimated weights differ substantially from those proposed here, the index as formulated in this article should be restructured or abandoned rather than adjusted post hoc to preserve it. A conceptual proposal is useful only in so far as it is capable of being refuted.

## 4. Discussion

### 4.1. Rationale and Positioning of the Index

The DWI is proposed as a structured response to a specific and, in our view, under-addressed gap: the absence of an instrument that combines the pulmonary, cardiac, inflammatory and metabolic dimensions of COPD into a single quantity computable from data already present in the routine clinical record. The limitations of this proposal, and in particular the absence of any empirical testing, are set out in Section 4.6 and are not minor. Our framework is consistent with the concept of COPD as a systemic condition in which outcomes are not determined solely by airflow limitation, but by the accumulation of extrapulmonary vulnerabilities. The DWI is proposed as one way of operationalising a shift from organ-centred classification to patient-centred risk stratification; whether it does so usefully remains to be shown.

Compared with conventional approaches primarily anchored to FEV_1_ and symptom burden, the DWI integrates pulmonary instability with systemic inflammation, cardiopulmonary stress, metabolic dysregulation, functional limitation, and exposure factors—domains that commonly co-exist and jointly shape prognosis [3,20,83].

A key clinical challenge in COPD is identifying instability before overt deterioration. Although prior exacerbations remain central, drivers of worsening are frequently extrapulmonary. Cardiac decompensation may mimic or amplify respiratory worsening, and dysglycaemia has been associated with lower lung function [53] and, in critically ill patients with COPD, with higher in-hospital mortality [57]. By combining biomarkers (NT-proBNP, CRP) with functional and metabolic measures (NYHA class, HbA1c, BMI), the DWI is intended to capture “hidden” systemic vulnerability; whether it allows earlier recognition of patients at risk despite apparently stable respiratory status is a hypothesis to be tested [52,56,57].

### 4.2. Influence of Pharmacological Treatment on the Components of the Index

Four of the ten components of the DWI are directly modifiable by drugs that patients with COPD frequently receive, and this has two distinct consequences that the current version of the index does not resolve. The first is that treatment alters the measured value: loop diuretics, renin–angiotensin system inhibitors, angiotensin receptor–neprilysin inhibitors, beta-blockers and sodium–glucose cotransporter 2 inhibitors all reduce NT-proBNP concentrations, and sacubitril/valsartan in particular lowers NT-proBNP while raising BNP; statins reduce CRP through an effect that is partly independent of lipid lowering; insulin, metformin and glucagon-like peptide 1 receptor agonists reduce HbA1c; and glucagon-like peptide 1 receptor agonists, systemic corticosteroids and nutritional supplementation all modify body mass index, in opposite directions. The second consequence is more subtle and more important: the prognostic meaning of a given value may differ according to whether it was obtained on or off treatment. A patient whose NT-proBNP is 400 pg/mL while receiving optimal heart failure therapy is not prognostically equivalent to an untreated patient with the same concentration, and an index that scores only the value cannot distinguish them.

We consider this a genuine limitation rather than an oversight that can be repaired at the conceptual level, since resolving it requires data. Three approaches will be evaluated at the derivation stage: recording concomitant therapy as a covariate and testing it formally as an effect modifier of the individual items; restricting computation of the index to values obtained under stable therapy for at least three months, which is the pragmatic solution we favour for a first prospective evaluation; or scoring the treated state itself, for instance treated versus untreated cardiac or metabolic comorbidity, rather than the biomarker value alone. Until this question is settled, DWI values obtained during or immediately after a change in therapy should not be compared with previous values in the same patient.

### 4.3. Longitudinal Behaviour and Use of the Index for Monitoring

COPD alternates periods of clinical stability with exacerbations, and every component of the DWI is time-varying. NT-proBNP and CRP in particular rise several-fold during an exacerbation and may require weeks to return to baseline, while FEV_1_ and CAT recover incompletely in a substantial proportion of patients. A DWI computed during an exacerbation therefore measures a different quantity from one computed in the stable state, and the two must not be pooled or compared.

We propose that the index be computed in the clinically stable state, defined as at least six weeks from the resolution of an exacerbation and with unchanged maintenance therapy, and that it be recomputed, in the prospective evaluation, at intervals linked to the provisional risk band: annually at low risk, every six months at moderate risk, every three to four months at high risk, and after every exacerbation at very high risk. The index is intended to estimate the risk of the primary composite outcome defined in Section 3.5—severe exacerbation, all-cause hospitalisation or all-cause death—over the 12 months following each assessment; when it is recalculated at the interval indicated by the risk band, each new value refers to the risk over the subsequent interval, so that the prognostic horizon advances with the patient rather than being fixed at baseline. This 12-month horizon is the primary prediction window pre-specified for the validation programme (Section 3.5), against which the discrimination and calibration of the index will be tested. Within this framework three distinct quantities are of potential interest and are to be tested separately: the baseline value, the trajectory across repeated measurements, and the extent of recovery after an exacerbation. Whether the trajectory carries prognostic information beyond the baseline value is an empirical question that a single-timepoint conceptual model cannot answer, and the repeated-measures component of the programme described in Section 3.5 is designed specifically to address it. We note that a composite index may in principle be less responsive than its most dynamic component, since a marked change in one item can be diluted by the stability of the remaining nine; reporting the domain sub-scores alongside the total mitigates, but does not eliminate, this problem.

### 4.4. Rationale for the Parameters and Comorbidities Included and Excluded

The comorbid dimensions represented in the DWI—cardiac strain, systemic inflammation and dysglycaemia—were not selected because they are the only ones that matter in COPD. Chronic kidney disease, anaemia, osteoporosis, depression and anxiety, sarcopenia, gastro-oesophageal reflux and lung cancer are all highly prevalent in this population and all affect prognosis; in the COTE index several of them carry mortality weights equal to or greater than those attributed to cardiac disease [30]. Their exclusion was governed by three criteria, which we state explicitly so that they can be contested. First, the parameter had to be available as a number in the routine record of an unselected COPD outpatient, which excludes conditions requiring dedicated instrumental assessment such as osteoporosis (densitometry) or lung cancer (low-dose computed tomography). Second, it had to be measurable on a continuous or ordinal scale rather than as a binary diagnosis, so that within-patient change could be tracked over time; this excludes categorical diagnoses such as depression or previous malignancy unless a validated scale is incorporated. Third, it had to be non-redundant with parameters already included.

Exercise capacity and physical performance require separate consideration. The six-minute walk distance is a core component of the BODE index and a robust predictor of mortality in COPD [25], and measures of balance and physical function such as the Timed Up and Go test and the Berg Balance Scale capture frailty, sarcopenia and fall risk, which are common in older patients and plausibly influence hospitalisation and survival. These measures were not included among the ten core items because they do not meet the first criterion in many outpatient settings: a standardised six-minute walk test requires a dedicated corridor, trained staff and two tests per session [81], and balance tests are rarely recorded during routine respiratory follow-up. NYHA class reflects functional limitation only coarsely and is not a substitute for them. Rather than omit them, we pre-specify these measures as candidate predictors for an extended version of the index, to be evaluated in centres where they are collected routinely: the derivation phase will test whether their addition improves discrimination and reclassification over the core index, and whether simpler surrogates of lower burden, such as gait speed or the one-minute sit-to-stand test, retain the same information (Section 3.5).

Applying these criteria, the most defensible criticism concerns chronic kidney disease. Estimated glomerular filtration rate is universally available, continuous, strongly prognostic in COPD, and it is also the principal non-cardiac determinant of NT-proBNP concentrations, which means that its omission both discards information and leaves a known confounder of an included item unaddressed. We therefore regard the addition of estimated glomerular filtration rate to the Cardio–Systemic domain as the single most likely modification of the index at the derivation stage, with haemoglobin as the second candidate, since anaemia is common, continuously measured and independently associated with mortality in COPD. Rather than expanding the model speculatively and without data, the derivation protocol will collect these variables and test their incremental value formally, so that the final composition of the index is determined empirically rather than by narrative selection.

### 4.5. Relationship with the Treatable Traits Framework

The DWI also aligns with the view of COPD as a heterogeneous syndrome with overlapping phenotypes and treatable traits, supporting a shift from organ-centred classification to patient-centred risk stratification [72]. Whether translating the score into graded risk categories can usefully inform decisions on follow-up intensity and multidisciplinary interventions is a hypothesis for future evaluation.

The treatable traits framework proposes that patients with chronic airway disease be characterised by identifiable, measurable and treatable pulmonary, extrapulmonary and behavioural traits rather than by diagnostic labels [84]. The correspondence with the DWI is deliberate: each of the ten components maps onto a trait for which an intervention exists—airflow limitation onto bronchodilation, exacerbation history onto inhaled and, in selected phenotypes, biological therapy, symptom burden onto pulmonary rehabilitation, cardiac strain onto cardiological assessment and heart-failure therapy, systemic inflammation onto cardiovascular risk reduction, dysglycaemia onto metabolic treatment, low or excessive body mass onto nutritional intervention, and current smoking onto cessation support. The index differs from a trait checklist in that it quantifies the aggregate burden and orders the traits by provisional weight, which a checklist does not do; conversely, a checklist preserves the identity of the individual trait, which a single summary score necessarily loses. For this reason, we recommend that the four domain sub-scores, and not only the total, be reported whenever the index is used: two patients with a DWI of 50 driven respectively by the Pulmonary–Symptomatic domain and by the Cardio–Systemic domain have the same score but might call for different interventions, and an index that obscured this distinction could be misleading.

### 4.6. Limitations

The primary limitation of this work is the absence of empirical testing. A prognostic index requires a derivation cohort, formal estimation of its coefficients, assessment of discrimination and calibration, and confirmation in an independent validation cohort; none of these steps has been performed here, and the predictive value and clinical utility of the DWI are consequently unproven. We regard this as a limitation of a degree that constrains how the present work should be read: it proposes and specifies an index, and it does not establish one.

Seven further limitations follow. First, the weights, although obtained through a pre-specified and transparent procedure (Section 2.12), remain heuristic, judgement-based and literature-anchored: five of the ten parameters could not be anchored to an adjusted estimate matching the categories used by the index, and the estimates that were available derive from studies differing in population, outcome definition and adjustment set and were not pooled by formal meta-analysis. The ordinal mapping provides at most a provisional ordering and does not establish the relative predictive contribution of the parameters; the age weight rests on estimates for composite indices and the NYHA weight on a proxy. The weights should therefore be read as expert-informed specifications rather than as established prognostic contributions. Second, several components are mutually correlated, so that the additive model may over-represent the cardiac and inflammatory constructs. Third, the categorisation of continuous variables into three ordinal levels discards information and introduces threshold effects at the cut-points, whereby a clinically trivial difference across a boundary changes the score while a larger difference within a category does not. Fourth, the index does not account for concomitant treatment (Section 4.2), for renal function, for exercise capacity and physical performance, or for comorbidities outside the four domains (Section 4.4). Fifth, the boundaries of the risk bands (25, 50 and 75) are equidistant divisions of the scale rather than empirically derived thresholds, and the follow-up strategies attached to them are expert opinion rather than evidence-based recommendations, as already stated in Section 3.3. Sixth, the proposed interpretation of longitudinal changes is provisional and no minimal clinically important difference has been established (Section 3.2). Seventh, and most fundamentally, the benefit of weighting itself is unproven: in the derivation of the BODE index, differential weighting of the components produced no gain in predictive value [25], and although that finding was obtained in an index built exclusively from pulmonary and functional variables, it establishes that a weighted model must demonstrate superiority over the unweighted sum of the same items rather than assume it. Three further points are recorded for completeness. First, biological sex is not scored, because a fixed binary attribute would make the maximum attainable score depend on sex; it is retained as a stratification covariate for the derivation phase (Section 2.11). Second, the collapse of the CAT from four to three categories sacrifices the distinction between the high and very high symptom-impact bands (Section 2.13). Third, because the NT-proBNP thresholds are age-stratified, age is partially represented twice in the index, and the non-linear BMI–mortality relationship means that class-III obesity, in which the protective association is lost, is not distinguished; both are pre-specified for empirical resolution at the derivation stage. A further group of limitations concerns the face validity of the fixed ordinal scoring. Because every parameter is scored on the same capped three-level scale and carries a fixed weight, the current specification cannot reproduce the steep, and probably non-linear, rise in risk that accompanies extreme values of some variables. Advanced age is the clearest example: a single upper age category combined with a low weight means that a patient in the tenth decade is separated from a much younger patient by only a few points on the 0–100 scale, which understates the near-certain excess risk at that age. The three-level body mass index item likewise assigns the same lowest-risk score across the whole range from normal weight to class-III (morbid) obesity, and therefore does not capture the loss, above approximately 40 kg/m^2^, of the protective association described by the obesity paradox. HbA1c carries the lowest weight, assigned because no adjusted estimate matching the categories used by the index could be identified in the COPD-specific literature, so that the difference between well-controlled and severely uncontrolled diabetes exerts only a marginal effect on the total score and, in all probability, under-represents the associated cardiovascular and mortality risk. We regard these observations not as defects to be corrected by ad hoc reweighting—which would reintroduce precisely the instability that must be avoided—but as concrete demonstrations that literature-anchored a priori weights cannot be finalised by inspection and must be estimated from data. Each is therefore pre-specified as an explicit analysis of the derivation phase (Section 3.5): a data-driven and potentially non-linear function of age; a non-linear body-mass-index term that allows the protective association to be lost at class-III obesity; and empirical re-estimation of the HbA1c weight across the full range of glycaemic control.

It follows that the DWI must not be used to guide the management of individual patients until the programme set out in Section 3.5 has been completed, and we ask that any subsequent use of this framework state this restriction explicitly.

## 5. Conclusions

The DWI is proposed as a multidimensional framework, based on routinely available data, integrating Pulmonary–Symptomatic, Cardio–Systemic, Metabolic–Nutritional and Demographic–Exposure domains and intended to estimate global risk in COPD. Whether moving beyond FEV_1_-centred stratification in this way can support more personalised monitoring and integrated management is a hypothesis that the programme described here is designed to test. Derivation and internal and external validation are required before any clinical implementation, and until then the index should be used only in research settings. At this stage the DWI should therefore be understood as a protocol for an index rather than as an index ready for use: it is a hypothesis, formulated so as to be testable and, if the evidence so indicates, refutable.

## Figures and Tables

**Table 4:** Worked examples. Three hypothetical patients scored through the Disease Weighted Index (DWI). Each cell reports the observed value, the corresponding ordinal score in parentheses and the weighted contribution. The patients are illustrative constructions; no clinical data were used.

Parameter (Weight)	Patient 1	Patient 2	Patient 3
FEV_1_ (0.12)	62% (1) = 0.12	68% (1) = 0.12	41% (2) = 0.24
Exacerbations (0.08)	1 moderate (1) = 0.08	0 (0) = 0	2, 1 hospitalised (2) = 0.16
CAT (0.12)	14 (1) = 0.12	9 (0) = 0	24 (2) = 0.24
NT-proBNP (0.16)	90 pg/mL (0) = 0	620 pg/mL (2) = 0.32	1250 pg/mL (2) = 0.32
CRP (0.12)	2.1 mg/L (0) = 0	6.2 mg/L (2) = 0.24	4.2 mg/L (1) = 0.12
NYHA (0.12)	I (0) = 0	II (1) = 0.12	III (2) = 0.24
HbA1c (0.03)	5.9% (0) = 0	7.8% (2) = 0.06	7.9% (2) = 0.06
BMI (0.12)	26.4 (0) = 0	32.0 (0) = 0	22.4 (1) = 0.12
Age (0.08)	62 y (0) = 0	71 y (1) = 0.08	78 y (2) = 0.16
Smoking (0.03)	Former, 15 py (1) = 0.03	Former, 30 py (1) = 0.03	Current, 45 py (2) = 0.06
Weighted sum	0.35	0.97	1.72
DWI = (sum/1.96) × 100	17.9	49.5	87.8
Risk category	Low	Moderate	Very high
Cardio–Systemic sub-score	0 of 0.80 (0%)	0.68 of 0.80 (85%)	0.68 of 0.80 (85%)

**Table 5:** Provisional risk-stratification thresholds and hypothesised interpretation according to the proposed Disease Weighted Index (DWI) framework. Increasing DWI values are intended to indicate progressively higher disease burden and risk of adverse outcomes; this has not yet been tested.

DWI Range	Risk Category	Provisional Interpretation (Hypothesis to Be Tested)
0–25.0	Low risk	Low multidimensional disease burden, stable clinical status, low theoretical risk of exacerbations, hospitalisation and mortality.
>25.0–50.0	Moderate risk	Intermediate disease burden with early functional, symptomatic or systemic involvement; increased risk of future exacerbations.
>50.0–75.0	High risk	High multidimensional burden with frequent exacerbations and/or significant systemic and metabolic comorbidities; elevated risk of hospitalisation.
>75.0–100	Very high risk	Severe multidimensional disease burden with advanced respiratory and systemic involvement; very high risk of adverse events.

**Table 6:** Conceptual comparison between the proposed Disease Weighted Index (DWI) and the established multidimensional indices in COPD. BODE, body mass index, obstruction, dyspnoea, exercise capacity; ADO, age, dyspnoea, obstruction; DOSE, dyspnoea, obstruction, smoking, exacerbations; CODEX, comorbidity, obstruction, dyspnoea, exacerbations; COTE, COPD-specific comorbidity test.

Index	Components	Dimensions Covered	Primary Outcome	Evidential Status
BODE [25]	BMI, FEV_1_, mMRC dyspnoea, 6-min walk distance	Pulmonary, functional, nutritional	All-cause mortality	Derived and validated (validation cohort in the original study)
ADO [26,27]	Age, mMRC dyspnoea, FEV_1_	Pulmonary, demographic	3-year mortality	Derived and externally validated
DOSE [28]	mMRC dyspnoea, FEV_1_, smoking status, exacerbations	Pulmonary, exposure	Disease severity, healthcare utilisation	Derived and validated
CODEX [29]	Charlson comorbidity, FEV_1_, mMRC dyspnoea, exacerbations	Pulmonary, comorbidity (as diagnoses)	Mortality and readmission after hospitalisation	Derived and validated in post-discharge cohorts
COTE [30]	12 comorbidities weighted by mortality risk	Comorbidity only	All-cause mortality	Derived and validated; complementary to BODE
DWI (proposed)	FEV_1_, exacerbations, CAT, NT-proBNP, CRP, NYHA, HbA1c, BMI, age, smoking	Pulmonary, cardiac, inflammatory, metabolic, demographic	12-month composite of severe exacerbation, all-cause hospitalisation or all-cause death (pre-specified)	Not derived and not validated; conceptual proposal only

**Table 7:** Elements of the Disease Weighted Index (DWI) framework specified conceptually in the present work, and elements to be established empirically in the pre-specified derivation and validation programme.

Element	Specified in the Present Work (Conceptual)	To be Established Empirically (Phase)
Candidate predictors	Ten core items selected a priori (Section 2.1, Section 2.2, Section 2.3, Section 2.4, Section 2.5, Section 2.6, Section 2.7, Section 2.8, Section 2.9, Section 2.10 and Section 2.11)	Retention, re-weighting or removal by multivariable modelling; eGFR, haemoglobin, 6MWD, TUG and BBS tested as additional candidates (Phase 1)
Categorisation and cut-offs	Guideline-based three-level categories (Table 2 and Table 3); FEV_1_ reference standard (Section 2.13)	Continuous and non-linear functional forms (age, BMI, HbA1c, FEV_1_ z-score) compared with categorised versions (Phase 1)
Weights	Provisional, expert-informed weights from literature-anchored ordinal tiers (Table 1)	Joint re-estimation; comparison with the unweighted sum (Phase 1)
Overlapping constructs	Acknowledged; cardiac items grouped in one domain	Variance inflation factors, joint estimation, domain-level versus item-level models (Phase 1)
Prediction target and horizon	12-month composite of severe exacerbation, all-cause hospitalisation or all-cause death	Event rates; component-specific and 24-month analyses (Phases 1–2)
Risk categories	Provisional equidistant bands (Table 5)	Thresholds on calibrated predicted risk; decision curve analysis (Phase 2)
Management pathways	Expert-opinion hypotheses (Section 3.3, Figure 2)	Interventional evaluation only after validation (Phase 3)
Model performance	Not assessed	Discrimination, calibration and reclassification versus BODE and CODEX (Phases 1–2)
Longitudinal use and MCID	External anchors pre-specified; band crossing excluded as an anchor (Section 3.2)	Anchor- and distribution-based estimation; trajectory analysis (Phase 3)
Sample size and reporting	Minimum number of events stated	Riley criteria [80]; reporting according to TRIPOD + AI [78]

## Data Availability

The original contributions presented in this study are included in the article. Further inquiries can be directed to the corresponding author.

## Abbreviations

The following abbreviations are used in this manuscript:

COPD	Chronic obstructive pulmonary disease
DWI	Disease Weighted Index
FEV_1_	Forced expiratory volume in one second
CAT	COPD assessment test
NT-proBNP	N-terminal pro–B-type natriuretic peptide
CRP	C-reactive protein
NYHA	New York Heart Association
HbA1c	Glycated haemoglobin
BMI	Body mass index
CVD	Cardiovascular disease
GOLD	Global Initiative for Chronic Obstructive Lung Disease
BODE	Body mass index, airflow obstruction, dyspnoea, exercise capacity index
ADO	Age, Dyspnoea, Obstruction index
CODEX	Comorbidity, Obstruction, Dyspnoea, Exacerbations index
COTE	COPD-specific comorbidity test
DOSE	Dyspnoea, Obstruction, Smoking, Exacerbations index
eGFR	Estimated glomerular filtration rate
HR	Hazard ratio
MCID	Minimal clinically important difference
6MWD	Six-minute walk distance
BBS	Berg Balance Scale
GLI	Global Lung Function Initiative
NRI	Net reclassification improvement
TRIPOD	Transparent Reporting of a multivariable prediction model for Individual Prognosis or Diagnosis
TUG	Timed up and go test
VIF	Variance inflation factor
